# Deciphering the complete genome sequence of multidrug-resistant *Escherichia coli* strain Hakim RU_GHWS isolated from ladies' hall sewage water in Bangladesh

**DOI:** 10.1128/mra.00741-24

**Published:** 2024-09-16

**Authors:** Md. Arif-Uz-Zaman Polash, Nusrat Zahan, Muhib Ullah Khan, Subir Sarker, Tianyu Zhang, Md. Hakimul Haque

**Affiliations:** 1 Department of Veterinary and Animal Sciences, University of Rajshahi, Rajshahi, Bangladesh; 2 Biomedical Sciences & Molecular Biology, College of Public Health, Medical and Veterinary Sciences, James Cook University, Townsville, Queensland, Australia; 3 Australian Institute of Tropical Health and Medicine, James Cook University, Townsville, Queensland, Australia; 4 State Key Laboratory of Respiratory Disease, Guangzhou Institutes of Biomedicine and Health (GIBH), Chinese Academy of Sciences (CAS), Guangzhou, China; University of Maryland School of Medicine, Baltimore, Maryland, USA

**Keywords:** whole genome, sewage water, multidrug-resistant, *Escherichia coli*, Bangladesh

## Abstract

This report details the genome sequence of *Escherichia coli* strain Hakim RU_GHWS, isolated from sewage water. The assembled genome comprises 5.022 Mb with 77.675× coverage, depicting an average GC content of 50.50%. This genome contains 10 CRISPR arrays, 14 prophages, 65 antibiotic resistance genes, and 28 virulence factor genes.

## ANNOUNCEMENT

*Escherichia coli*, commonly found in the lower intestines of humans and animals, include strains that pose significant health risks (1). Sewage water increases antimicrobial resistance (AMR) due to dense bacterial environments and antibiotics, promoting horizontal gene transfer among bacteria (2). Contaminated water can spread resistant bacteria to drinking water, agriculture, and ecosystems, increasing human exposure (2). Multidrug-resistant *E. coli* in animals, humans, food, and environments highlight the need for AMR surveillance through the One Health approach to guide targeted public health interventions (3–6).

The Institute of Biological Sciences (IBScs) at the University of Rajshahi, Bangladesh, approved this study under Memo No. 56/321/IAMEBBC/IBScs. In September 2023, following standard procedures, we collected sewage water samples at the University of Rajshahi (24.3733°N, 88.6049°E). The samples were mixed thoroughly, transferred to sterile tubes, and transported to the laboratory. We then inoculated these samples on UTI agar (HiMedia, India) and incubated them aerobically at 37°C for 18–24 hours (6). *E. coli* was isolated using MacConkey (HiMedia, India) and eosin methylene blue agar (HiMedia, India), followed by staining and biochemical tests (7). Antibiogram study of the isolates was performed using the disk diffusion method (8), following CLSI guidelines (9). The strain presented resistance to gentamicin, amoxicillin, doxycycline, cephradine, ciprofloxacin, co-trimoxazole, and azithromycin. We cultured the isolated strain in nutrient broth (HiMedia, India) overnight at 37°C and then extracted its genomic DNA using the Qiagen DNA Mini Kit (QIAGEN, Hilden, Germany). The genomic DNA was enzymatically fragmented using the NEBNext dsDNA Fragmentase Kit (NEB, MA, USA), and size selection was carried out with SPRI beads (10). A sequencing library was prepared using the Nextera DNA Flex Library Preparation Kit (Illumina, San Diego, CA, USA), and the library was sequenced with 2 × 150 paired-end reads on the Illumina NextSeq2000 platform. Quality checks were performed using FastQC v0.11.7 (11). Raw reads (*n* = 4,517,090) were trimmed using Trimmomatic v0.39 (12), and genome assembly was conducted using Unicycler v0.4.9 (13). The annotation of the genome was carried out using PGAP v3.0 (14). The assembled genome was analyzed for antibiotic resistance genes (ARGs) using CARD v.3.2.4 with RGI v6.0.2 (15) and ResFinder v.4.1 (16), virulence factor genes using VFDB with VFanalyzer v.4.0 (17), pathogenicity index using PathogenFinder v.1.1 (18), sequence type using MLST v.2.0 (19), CRISPR arrays using CRISPRimmunity (20), and metabolic functional features using RAST v.2.0 (21). We used default parameters for all tools unless noted otherwise.

**Fig 1:**
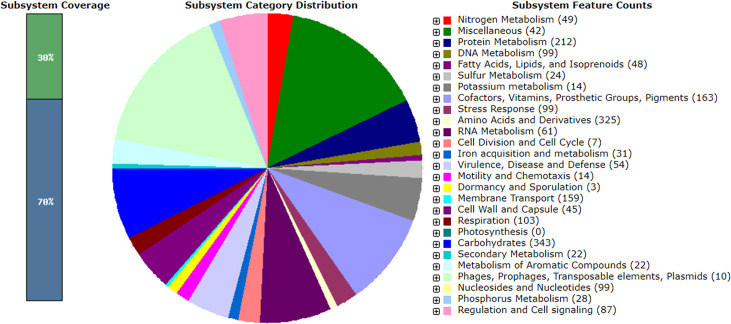
Metabolic functional features in the assembled genome of the *E. coli* Hakim RU_GHWS in SEED viewer. The 30% coverage indicates the completeness of functional roles within a specific subsystem across different genomes.

The features of the draft genomes are chronicled in Table 1. Notably, 65 ARGs, 28 virulence genes, and 6 plasmids [IncFIA(HI1), IncFIB(pB171), IncFII, IncHI1A, IncHI1B(R27), and IncX1] were predicted. MLST classified the genome as sequence type unknown but nearest to 999, and the PathogenFinder tool specified a pathogenicity index of 0.913. The genome exhibited 10 CRISPR arrays with 16 signature genes (*cas3, csa3, cas2, cas1, cas6e, cas5, cas7, cse2gr11, cas8e, TnsC, DinG, DEDDh, WYL, PD-DExK, c2c9_V-U4,* and *cas3HD*) and 14 prophages. RAST analysis uncovered 373 subsystems comprising 5,083 genes with 30% coverage (Fig. 1).

**TABLE 1:** Genomic elements of the *E. coli* strain Hakim RU_GHWS

Elements	Values
Genome size	5,022,442 bp
Genome coverage	77.675×
G + C content	50.50%
Number of contigs	271
Contig L50	28
Contig N50	55,646 bp
Total genes	4,976
Coding sequences	4,918
Coding genes	4,733
RNA genes	58
tRNAs genes	48
rRNAs genes	2
tmRNAs gene	1
ncRNAs genes	8
Pseudo genes	185
Genes assigned to SEED subsystems	5,083
Number of subsystems	373

## Data Availability

The study on E. coli strain Hakim RU_GHWS, conducted using the WGS shotgun approach, was submitted to NCBI/GenBank, and the assembly was deposited under the accession number JBEHGP000000000. The pertinent data, including the original readings, were stored with BioProject accession number PRJNA1101920, BioSample accession number SAMN41006273, and SRA accession number SRR28733676. The specific version mentioned in this document is labeled as JBEHGP000000000.1.
